# Why Are Babies Dying in the First Month after Birth? A 7-Year Study of Neonatal Mortality in Northern Ghana

**DOI:** 10.1371/journal.pone.0058924

**Published:** 2013-03-19

**Authors:** Paul Welaga, Cheryl A. Moyer, Raymond Aborigo, Philip Adongo, John Williams, Abraham Hodgson, Abraham Oduro, Cyril Engmann

**Affiliations:** 1 Navrongo Health Research Centre, Ghana Health Service, Navrongo, Ghana; 2 Global REACH and the Department of Medical Education, University of Michigan Medical School, Ann Arbor, Michigan, United States of America; 3 School of Public Health, University of Ghana, Legon, Ghana; 4 Department of Paediatrics and Maternal Child Health, Schools of Medicine and Public Health, University of North Carolina, Chapel Hill, North Carolina, United States of America; 5 Jeffrey Cheah School of Medicine and Health Sciences, MONASH University, Sunway Campus, Malaysia; University of Massachusetts Medical School, United States of America

## Abstract

**Objectives:**

To determine the neonatal mortality rate in the Kassena-Nankana District (KND) of northern Ghana, and to identify the leading causes and timing of neonatal deaths.

**Methods:**

The KND falls within the Navrongo Health Research Centre’s Health and Demographic Surveillance System (HDSS), which uses trained field workers to gather and update health and demographic information from community members every four months. We utilized HDSS data from 2003–2009 to examine patterns of neonatal mortality.

**Results:**

A total of 17,751 live births between January 2003 and December 2009 were recorded, including 424 neonatal deaths 64.8%(275) of neonatal deaths occurred in the first week of life. The overall neonatal mortality rate was 24 per 1000 live births (95%CI 22 to 26) and early neonatal mortality rate was 16 per 1000 live births (95% CI 14 to 17). Neonatal mortality rates decreased over the period from 26 per 1000 live births in 2003 to 19 per 1000 live births in 2009. In all, 32%(137) of the neonatal deaths were from infections, 21%(88) from birth injury and asphyxia and 18%(76) from prematurity, making these three the leading causes of neonatal deaths in the area. Birth injury and asphyxia (31%) and prematurity (26%) were the leading causes of early neonatal deaths, while infection accounted for 59% of late neonatal deaths. Nearly 46% of all neonatal deaths occurred during the first three postnatal days. In multivariate analysis, multiple births, gestational age <32 weeks and first pregnancies conferred the highest odds of neonatal deaths.

**Conclusions:**

Neonatal mortality rates are declining in rural northern Ghana, with majority of deaths occurring within the first week of life. This has major policy, programmatic and research implications. Further research is needed to better understand the social, cultural, and logistical factors that drive high mortality in the early days following delivery.

## Introduction

Although child mortality is declining worldwide, an estimated 8.8 million children still die every year before their fifth birthday [1]. More than 40% of these children die within 28 days after birth (considered the “neonatal period”), [1] a burden borne disproportionately by low- and middle-income countries [2].

Sub-Saharan Africa has among the highest neonatal mortality rates in the world, yet some of the weakest health and vital registration systems[3]–[6]. Neonatal mortality rates are typically estimated using complex statistical modelling, small hospital-based studies, or nationally representative demographic and health surveys that use cluster-level sampling of live births [7]. As a result, neonatal mortality rates are often underestimated in developing country settings [4], [8], [9].

In Ghana, published neonatal mortality rates have ranged from fewer than 15 per 1000 live births [10] to more than 30 per 1000 live births [11]. Without a clear sense of the true burden of neonatal deaths and the aetiology of those deaths, planning appropriate interventions is extremely difficult.

This research sought to address these challenges by relying upon a well-established demographic surveillance system to collect primary data on all neonatal deaths in the Kassena-Nankana District of the Upper East Region in northern Ghana between 2003 and 2009. The Navrongo Health and Demographic Surveillance System (NHDSS) was instituted and managed by the Navrongo Health Research Centre (NHRC) to collect longitudinal data on births, deaths, pregnancies, marriages and migration using trained field workers who visit all households in the area three times a year to update both individual and household information[12]–[14]. Using data from the NHDSS, this study aimed to determine not only the neonatal mortality rate for the study area, but also to determine the leading causes of death and to examine the timing of deaths within the first month after delivery.

## Methods

### Ethics Statement

This study was reviewed and approved by the institutional ethics review committees of the Navrongo Health Research Centre, the University of North Carolina at Chapel Hill and the University of Michigan.

We sought verbal consent from household heads or any other elderly member of the household to update the health and demographic information of household members every four months including verbal autopsies for the dead as part of the operations of the Navrongo Health and Demographic Surveillance System (NHDSS). As a health and demographic surveillance system with frequent visits to households to update health and demographic information, verbal consent was deemed appropriate for monitoring of the demographic events which requires routine frequent visits to households. From 2011, written consent is required before the conduct of verbal autopsies for the dead. The information given out to respondents during the consent process is documented and field workers have been adequately trained to administer the consent. The consent process was approved by the Institutional Review Board of the Navrongo Health Research Centre.

### Study Setting

We conducted this study in the Kassena-Nankana District (KND) of the Upper East region of northern Ghana. The KND includes a population of approximately 152,000 residents who live within the catchment area of the NHDSS. The study area is typical of many rural areas in sub-Saharan Africa with the majority of inhabitants being subsistence farmers who live in small, scattered settlements. There are two main ethnic groups, the Kassenas and the Nankanis with a combined fertility rate of 3.8.

One district hospital in the KND draws from six smaller health centres and one private clinic in the study area. In addition, there are more than 30 Community Health Compounds (CHCs) which provide basic health care services in the area.

### Registration of Pregnancies, Deliveries and Deaths

Fieldworkers with a minimum of a high school education are trained for at least three weeks by NHRC in community entry and data collection procedures. Fieldworkers identified and registered all pregnancies, deliveries and deaths in the study area during their routine household visits, conducted once every four months. Community key informants, who are selected members of the community and trained by NHRC, complement the efforts of fieldworkers by registering all pregnancies, births and child deaths that occur in their communities. This is to ensure the timely capture of all demographic events, especially neonatal deaths. Each registered pregnancy was monitored until completion and categorized as resulting in a live birth or stillbirth, a miscarriage, an abortion, or a migration if the pregnant woman moved out of the study area. We determined gestational age using the mother’s last menstrual period which was established at the time of registering the pregnancy. Our operational definition of stillbirth at the population level is fetal death at 28 weeks gestation or more with no signs of life at birth, i.e. no breathing, no heart rate and no movement. Unique identifiers linked maternal health and demographic data to infant data. Eighty-five percent of all pregnancies were registered by week 28. Verbal autopsies (VA) were conducted on all infant deaths that were identified by the field team.

### Determining Cause of Death

We used validated VA tools to assess cause of death among neonates[15]–[17]. The VA instruments include a battery of questions as well as a section for verbatim narrations of the circumstances leading to the death. Experienced field supervisors trained by the NHRC conducted the interviews with the neonate’s immediate caregiver. VA interviews were typically conducted approximately 3 months after deaths occurred. This interval was chosen to minimize the risk of additional emotional trauma for respondents by interviewing them too soon after a neonatal death, and to decrease the likelihood of recall bias [18], [19].

Data collection was rigorously supervised, and 5% of interviews were repeated for quality assurance. When discrepancies were detected, interviews were repeated.

Three physicians each reviewed the VA forms and assigned an underlying cause of death (COD) corresponding to the 3-digit code of the international statistical classification of diseases and health-related problems [20]. If at least two agreed on the underlying COD, a diagnosis was established. Where there was disagreement among all three, the form was submitted to two additional physicians for review. Where there was VA information but no consensus could be reached regarding underlying COD, the case was declared undetermined. Where little or no information was available to enable an assignment of COD, the diagnosis was declared unknown.

### Statistical Methods

We used STATA 11.2 for all analysis. Frequencies and descriptive statistics were calculated for both maternal and neonate characteristics. A wealth index was computed using principal component analysis (PCA) from household assets as an estimate of household socioeconomic status. The household assets in the analysis included more than 25 separate items, from large assets (e.g., land and car ownership) to smaller household items (e.g., radio, fan ownership). Factors potentially associated with neonatal mortality rates were grouped into three domains: maternal characteristics; delivery location; and infant characteristics. Unadjusted and adjusted odds ratios with 95% confidence intervals were computed to assess the relationship between neonatal mortality and selected variables. Reference categories were defined as those usually associated with the lowest neonatal mortality rates. All variables found to be significant in bivariate analysis were then included in a multivariate logistic regression model, and adjusted odds ratios with 95% confidence intervals were calculated.

## Results

**Figure 1:**
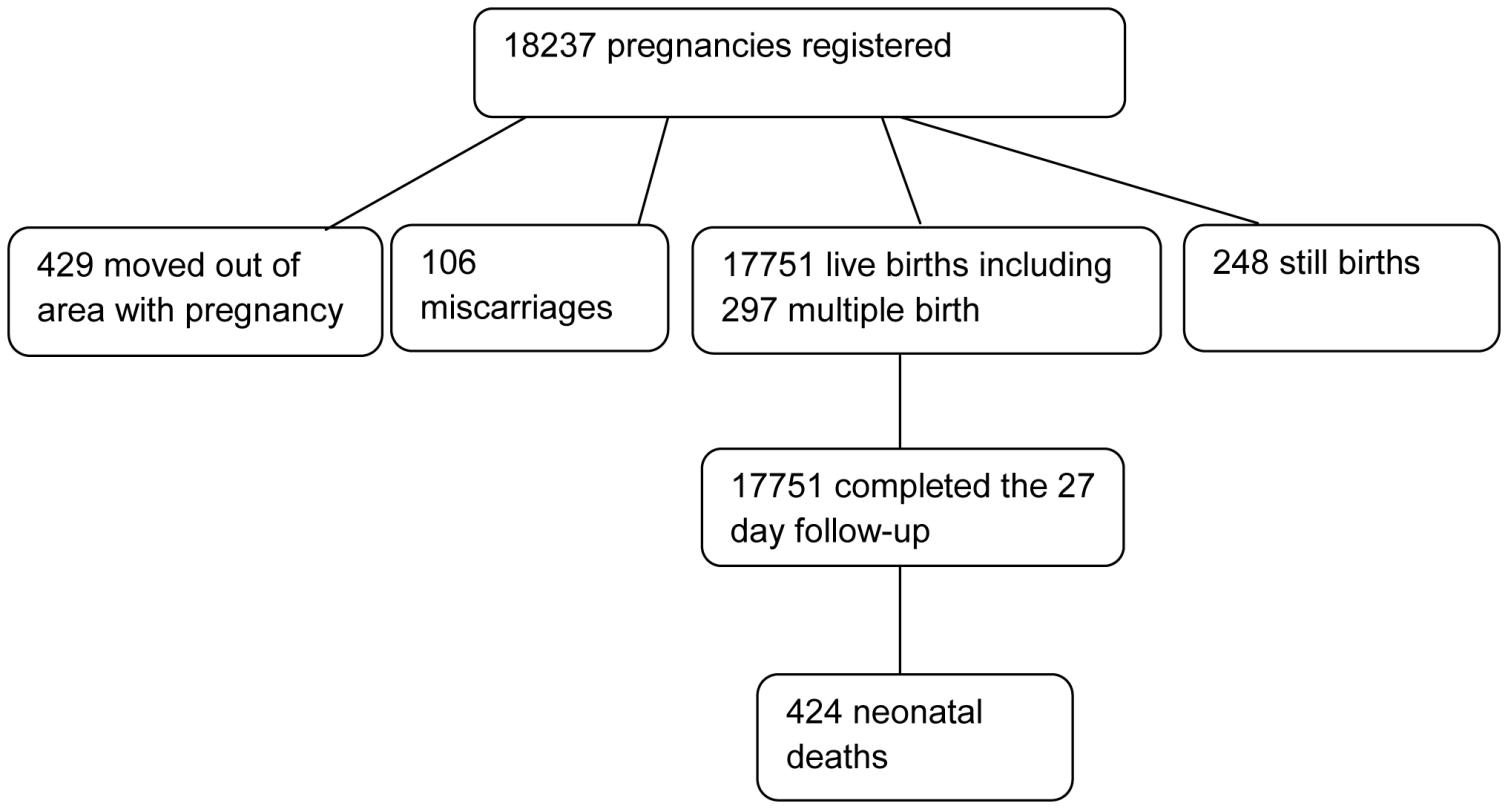
Study population of pregnancies registered in Navrongo HDSS: 2003–2009.

Between January 2003 and 2009, there were 18,237 pregnant women enrolled. In total, 429 mothers moved out of the study area before delivery, and there were 106 miscarriages and 248 stillbirths. The analysis was limited to children whose pregnancies were previously registered and monitored until completion. Among the pregnancy outcomes recorded, there were 17,751 live births including 297 multiple births. (See Figure 1.).

There were 424 neonatal deaths, 275 (64.8%) of which occurred in the first week of life. The neonatal mortality rate for the period was 24 per 1000 live births (95% CI 22 to 26) and the early neonatal mortality (ENM) rate was 16 per 1000 live births (95% CI 14 to 17). Neonatal mortality rates decreased over the 7-year period from 26 per 1000 live births in 2003 to 19 per 1000 live births in 2009 (See Table 1).

**Table 1:** Neonatal Mortality Rates and Leading Causes of Deaths in Kassena-Nankana District, Ghana (2003–2009).

	2003	2004	2005	2006	2007	2008	2009	Total
**Live Births**	2,935	2,824	2,577	2,465	2,605	2,421	1,924	17,751
**Number of Deaths(NMR/1000)**	77 (26.2)	66 (23.4)	74 (28.7)	49 (19.9)	55(21.1)	66(27.3)	37 (19.2)	424(23.9)
**Infective causes (CSNMR)**	33 (11.2)	22 (7.8)	22 (8.5)	21 (8.5)	10(3.8)	15(6.2)	14 (7.3)	137(7.7)
**Birth injury (CSNMR)**	13 (4.4)	15 (5.3)	11 (4.3)	11 (4.5)	17(6.5)	13(5.4)	8 (4.2)	88(5.0)
**Prematurity (CSNMR)**	18 (6.1)	13 (4.6)	14 (5.4)	7 (2.8)	10(3.8)	12(5.0)	2 (1.0)	76(4.3)
**Other disorders related to perinatal period (CSNMR)**	0	1 (0.4)	0	5 (2.0)	14(5.4)	19(7.8)	6 (3.1)	4 (2.5)
**Infanticide (CSNMR)**	2 (0.7)	3 (1.1)	2 (0.8)	2 (0.8)	0	0	0	9 (0.5)
**Undetermined/incomplete coding**	11 (3.7)	12 (4.2)	25 (9.7)	3 (1.2)	4(1.9)	7(2.9)	7 (3.6)	69 (3.9)

CSNMR – Cause-Specific Neonatal Mortality Rate.

Approximately 68% of all deliveries were full term (>36 weeks gestation) and 59% of deliveries occurred at home over the 7-year period. Slightly less than 10% of deliveries occurred to teenagers, and 26% of mothers were older than 35 at the time of delivery. More than 90% of mothers had either no formal education or education that ended after primary or junior secondary school, and 89% were either married or cohabitating. More than 70% of women had at least one antenatal care visit at the time of registering the pregnancy, and 96.5% of deliveries were singleton births. The mortality rate for singletons was 22 per 1000 compared to 72.4 per 1000 live births for multiple births (p<0.0001). About 21% of births occurred to mothers delivering from their first pregnancy and 77% had one or more children before this delivery.

In all, 32% (137) of the neonatal deaths were from infections, 21% (88) from birth injury and asphyxia and 18% (76) from prematurity, making these three the leading causes of neonatal deaths in this region of northern Ghana. Birth injury and asphyxia are put under the same classification in this study and it includes babies who die as a result of damages to the tissues and organs of the child including intracranial injury (brain injury) during delivery. These babies do not cry or cannot breathe, or have a shrill cry or seizures shortly after birth and this is often a result of difficulties during childbirth.

**Figure 2:**
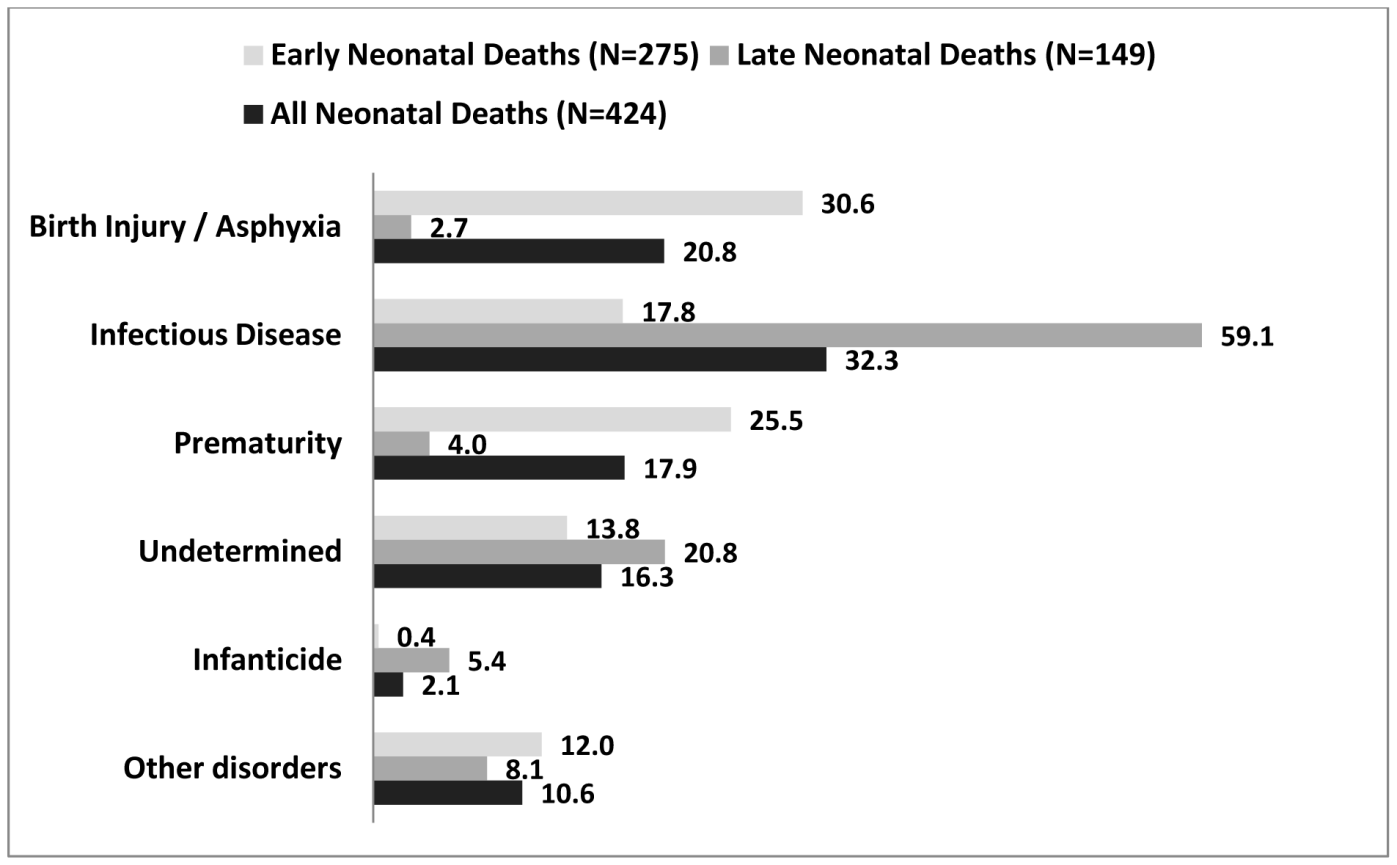
Causes of Early and Late Neonatal Deaths in Navrongo HDSS from 2003 to 2009*. *shown as a percent of early, late, all neonatal deaths.

**Figure 3:**
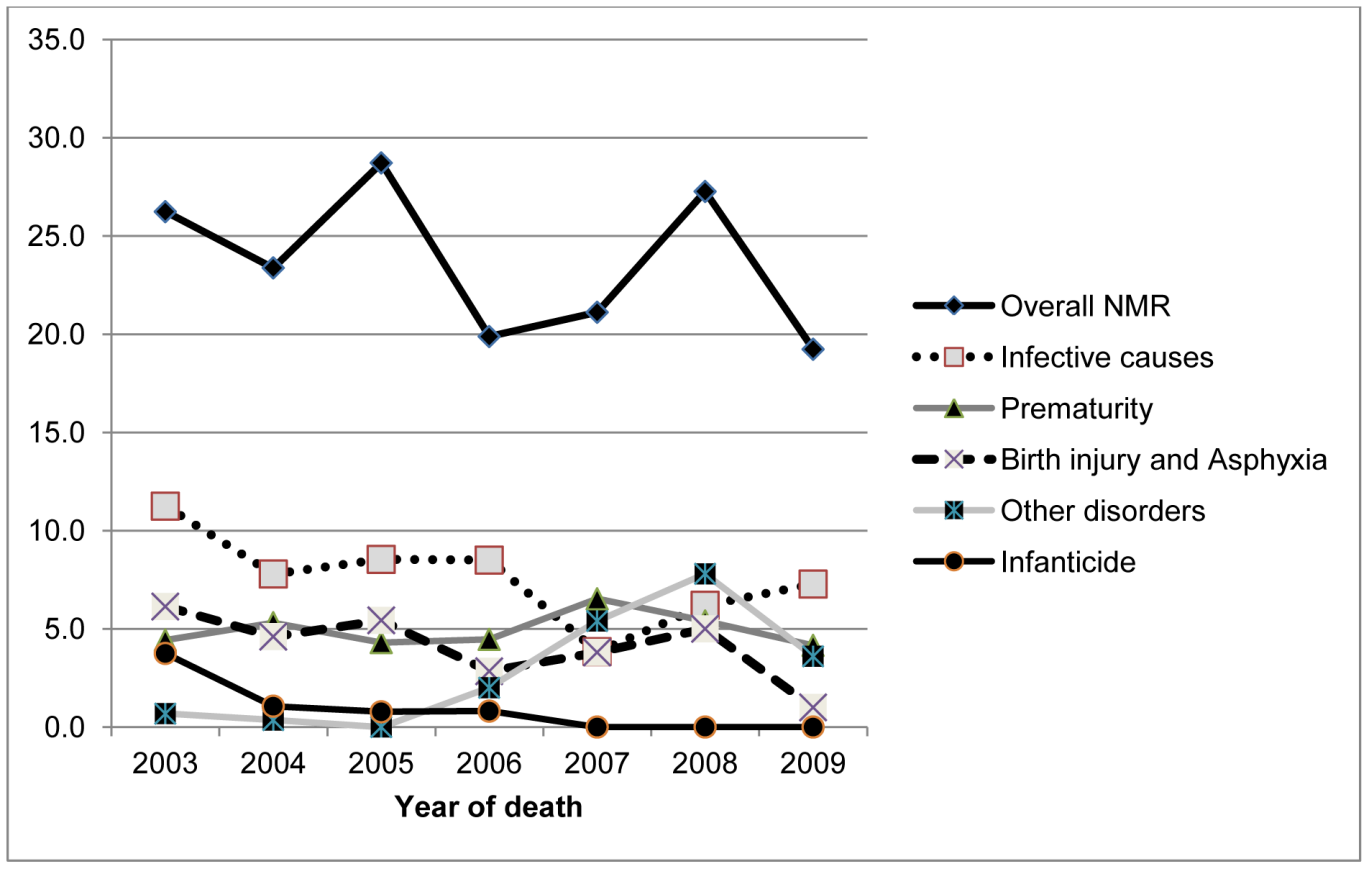
Trends in overall and cause-specific neonatal mortality rates in the Kassena-Nankana districts: 2003–2009 (n = 424).

Birth injury and asphyxia (31%) and prematurity (26%) were the leading causes of early neonatal deaths, while infection accounted for 59% of late neonatal deaths (See Figure 2). The main causes of death showed a decline in cause specific mortality rates over the period with increases for infections in 2008 and 2009 (See Figure 3). There was no neonatal death attributed to infanticide after 2007.

**Figure 4:**
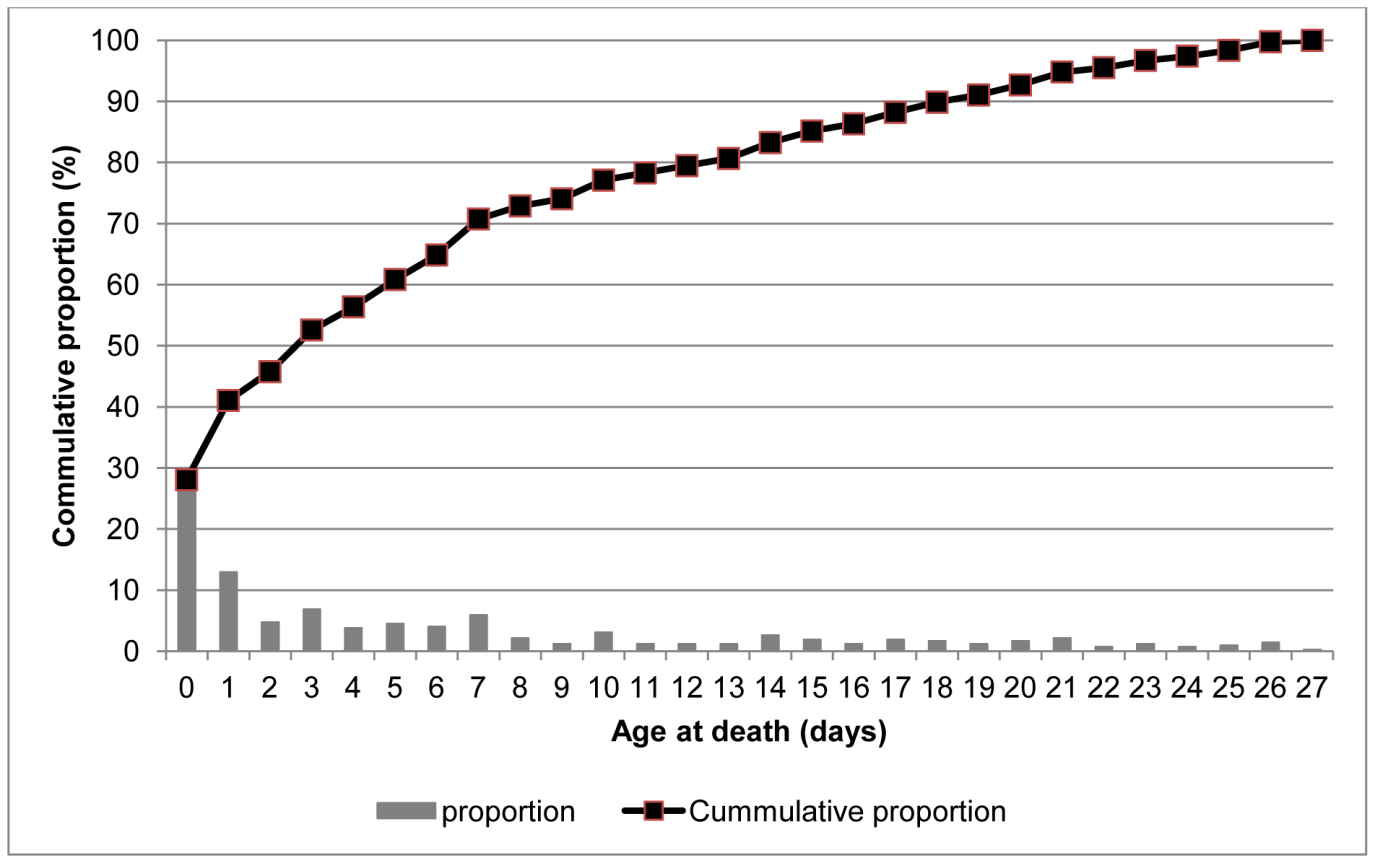
Timing of neonatal deaths in Navrongo HDSS: 2003–2009.

Figure 4 shows neonatal mortality rates by day of life. More than 28% of all neonatal deaths (119) occurred on the first day of life, 13% on the second day and approximately 5% on the third day. Nearly 46% of all neonatal deaths occurred during the first three postnatal days. There was a flattening of the cumulative proportion of neonatal deaths curve after the fourth day.

In bivariate analysis, maternal age younger than 20, being single, primiparity, prematurity, and multiple births were significant predictors of neonatal deaths (Table 2). Maternal age younger than 20 was associated with a 1.8-fold increased odds of neonatal death compared with mothers aged 20 to 34 years. Children delivered from first pregnancies had nearly twofold increased odds of death in the first 28 days compared with children from second or more pregnancies. Even though not statistically significant, male sex was associated with a 1.2-fold increased odds of death compared to females [OR = 1.19, 95% CI (0.98–1.45)].

**Table 2:** Demographic and health characteristics of mothers and neonates, as associated with neonatal deaths.

Category	Live born births	Neonatal deaths	Odds ratio	Adjusted OR
	n (%)	(NMR/1000 live births)	(95% CI)	(95% CI)
**Maternal age**				
<20	1,734 (9.8)	68 (39.2)	1.82 (1.38–2.39)*	NS
20–34	11,386 (64.1)	250 (22.0)	1	
35+	4,631 (26.1)	106 (22.9)	1.04 (0.83–1.31)	
**Educational status**				
No formal education	7,209 (40.6)	170 (23.6)	0.99 (0.68–1.43)	
Primary/JSS	9,051 (50.8)	215 (23.8)	1.00 (0.70–1.44)	
Secondary/tertiary	1,469 (8.3)	35 (23.8)	1	
Missing	58 (0.3)	4 (6.9)	3.03 (1.04–8.84)*	
**Presence of partner**				
Single	2,024 (11.4)	67 (33.1)	1.47 (1.13–1.92)*	NS
Married/cohabiting	15,727 (88.6)	357 (22.7)	1	
**First pregnancy**				
Yes	3,721 (21.0)	140 (37.6)	1.89 (1.54–2.32)*	1.52 (1.07–2.14)*
No	14,030 (79.0)	284 (20.2)	1	1
**Number of children**				
1	4,019 (22.6)	144 (35.8)	1.93 (1.54–2.42)*	NS
2–4	9,057 (51.0)	171 (18.9)	1	
5+	4,675 (26.4)	109 (23.3)	1.24 (0.97–1.58)	
**Perinatal care**				
No perinatal care	5,291 (29.8)	123 (23.2)	1	
One visit or more	12,460 (70.2)	301 (24.2)	1.04 (0.84–1.29)	
**Delivery location**				
Home/other	10,523 (59.3)	236 (22.4)	1	
Clinic/hospital	6,777 (38.2)	175 (25.8)	1.16 (0.95–1.41)	
Missing	451 (2.5)	13 (28.8)	1.29 (0.73–2.28)	
**Sex**				
Male	8,994 (50.7)	233 (25.9)	1.19 (0.98–1.45)	NS
Female	8,757 (49.3)	191 (21.8)	1	
**Gestation age**				
<32 weeks	1,596 (9.0)	99 (62.0)	3.39 (2.66–4.32)*	3.10 (2.43–3.96)*
32 to 36 weeks	4,079 (23.0)	94 (23.0)	1.21 (0.95–1.54)	1.11 (0.87–1.42)
>36 weeks	12,076 (68.0)	231 (19.1)	1	1
**Multiple**				
Yes	594 (3.3)	43 (72.4)	3.46 (2.50–4.80)*	3.91 (2.79–5.49)*
No	16,703 (94.1)	368 (22.0)	1	1
Missing	454 (2.6)	13 (28.6)	1.30 (0.75–2.29)	1.18 (0.67–2.08)
**Socioeconomic status**				
Poor	3,831 (21.6)	99 (25.8)	1.23 (0.90–1.67)	
Next poor	3,323 (18.7)	67 (20.2)	0.95 (0.68–1.33)	
Average	3,399 (19.1)	72 (21.2)	1	
Next rich	3,425 (19.3)	81 (23.6)	1.12 (0.81–1.45)	
Rich	1,875 (10.6)	53 (28.3)	1.34 (0.94–1.54)	
Missing	1,898 (10.7)	52 (27.4)	1.30(0.91–1.87)	
Total	17751 (100)	424 (23.9)		

* Indicates significance at 95% CI. Abbreviations: NS, not significant.

In multivariate analysis, multiple births, gestational age <32 weeks and first pregnancies conferred the highest odds of neonatal deaths. Infants from first pregnancies had a 1.5-fold increased odds of death. Multiple gestation was associated with a nearly fourfold increased odds of neonatal death and prematurity (gestational age <32 weeks) was associated with a threefold increase in the odds of neonatal death.

## Discussion

In this study we found that nearly two-thirds (64.8%) of the 424 neonatal deaths in the KND in northern Ghana occurred in the first week of life. Nearly half of all neonatal deaths (46%) occurred in the first three postnatal days. Leading causes of all neonatal deaths in this region were infections, birth injury and asphyxia, and prematurity, with early neonatal deaths most likely to result from birth injury and asphyxia and prematurity, and late neonatal deaths most likely to result from infections. In multivariate analysis, we found that multiple births, prematurity and first pregnancies conferred the highest odds of neonatal deaths.

Overall, we found a neonatal mortality rate in this region of 24 per 1000 live births (95% CI 22 to 26) during the study period. The rate fell from 26.2 neonatal deaths per 1000 live births in 2003 to 19 per 1000 live births in 2009. We also found an overall early neonatal mortality rate of 16 per 1000 live births (95% CI 14 to 17), a rate that had dropped from 17 per 1000 in 2003 to 11 per 1000 in 2009 (data not shown). Our study’s neonatal mortality rate for the period is higher than what was found in the 2008 Ghana Demographic Health Survey, which reported an overall neonatal death rate of 17 per 1000 live births from the Upper East region of Ghana [21] and the Child Health Epidemiology Reference Group (CHERG), which suggested Ghana had fewer than 15 neonatal deaths per 1000 live births [10]. However, a 2010 estimate by Lui et al suggested that Ghana had 28.1 neonatal deaths per 1000 live births across the country (uncertainty interval of 20.3 to 37.1) [22]. It is important to remember that the Ghana DHS analysis was based upon an unweighted random sample that included 500 infant deaths while the CHERG analysis was based upon a combination of civil registration data and sampled DHS data. Our findings include all neonatal deaths enrolled in NHDSS which were monitored from pregnancy until death between 2003 and 2009 and do not reflect sampling or statistical modelling.

Neonatal mortality rates have dropped in the KND since 2003, [3] a finding consistent with other literature [10]. However, the rate of drop in neonatal mortality is considerably less than that of under five mortality, both worldwide and in Ghana, resulting in the percentage of under five deaths that are attributable to neonatal mortality rising. [10]. Thus improvements in both under five and neonatal mortality are masking the disproportionate impact of deaths during the first 28 days of life in the developing world.

Our study showed that approximately 28% of all neonatal deaths occurred during the first day of life, 53% during the first three days of life, and 65% during the first week of life. These findings are consistent with other studies reported in the literature including those from Guatemala, India, Pakistan, Zambia and the Democratic Republic of Congo [9] [23]–[25]. It is clear that policies and programs that specifically target neonates during the first week of life are urgently needed to improve both neonatal and child mortality.

Our study identified prematurity, multiple births and first births to be significantly associated with increased odds of deaths after controlling for other factors in our multivariable model (see Table 2). Our findings are consistent with a similar study in Burkina Faso [26] as well as elsewhere in Ghana, Pakistan, and Democratic Republic of Congo [11], [24], [27]. Multiple births accounted for 3.3% of all births and 10% of all neonatal deaths in the study area. This may be due to the increased likelihood of multiple births being delivered preterm [28], thus increasing the odds of neonatal death. We also found that neonates born to primiparous women had increased odds of neonatal death even after controlling for maternal age and other confounding variables. Similar findings were observed in Central Africa [25] and in Ghana [29].

In our study, lower socioeconomic status (SES) did not confer increased odds for neonatal death. This is contrary to much of the existing literature that links neonatal mortality to slum residence, lower SES, or other indicators of poverty [30]–[32]. Our findings may in part be explained by the improvement in the health system in the KND during the study period, including the widespread implementation of Community-based Health Planning and Services (CHPS) which assigns nurses to regional community health centres to provide basic curative and preventive health care [14].

We also found that young maternal age (delivery before age 20) was not significantly associated with neonatal death rates in multivariate analysis, which is also contrary to existing literature [32]. Taken together, our data suggests that improving a region’s health system has the potential to offset the detrimental effect of poverty and teenage pregnancy on neonatal health.

We found the main causes of neonatal death in our study to be infections (32%), birth asphyxia and birth injuries (21%) and prematurity (18%). Most of the neonatal deaths from infections were due to septicaemia (86.9%), followed by acute lower respiratory infections (5.8%), meningitis (5.1%), anaemia (1.5%) and diarrhoea diseases (0.7%). Based on the detailed causes of death from infections, most of the deaths could be deduced to be from bacterial infections but a few of the gastro-enteritis cases could be viral infections.

Our findings are consistent with Black et al.’s systematic analysis which showed that in sub-Saharan Africa, infections, pre-term birth complications and birth asphyxia were the leading causes of neonatal death [1]. However, our results conflict with a study similar to ours conducted in neighboring Burkina Faso, which reported pre-term birth (42%) and infections (18%) to be the dominant causes of neonatal deaths [26]. In our study, neonatal deaths from infections as well as birth asphyxia and birth injuries appeared to decrease over the period, a finding we attribute to improvements in the health system and community’s access to health care between 2003 and 2009. Note however that neonatal deaths from prematurity appear to have stalled over the period.

We believe the results of our study have several important implications for research and practice. First, we found that neonatal mortality rates are improving in rural northern Ghana, a sparsely populated region with widespread poverty. While such a finding is encouraging, and may be attributable to increased community health services and a concerted effort to provide better healthcare for rural residents [13], [14], [29], our findings also suggest that a critical intervention period in this region is being missed: the first few days after birth. Further research is needed that explores what occurs in those first few days. Are women delivering at home and failing to seek post-partum care for their newborns? Are women delivering in facilities that are ill-equipped to handle complicated or premature deliveries, or are healthcare personnel insufficiently trained to resuscitate infants in need? Are women delivering in well-equipped facilities with well-trained personnel but returning home and failing to recognize danger signs in their infants? Poor infant feeding have also been shown to be associated with neonatal mortality especially in the first seven days of life in the study area [33]. Further research is warranted to address these and other causal questions.

Interestingly, the practice of infanticide appears to have stopped. Researchers have described the phenomenon of the “spirit child” [34] and how such infants, imbued with malevolent intent, brought deep misfortune to the family. These infants were perceived to be spirits “from the bush” and once labelled were taken “back to the bush” and left alone. In a 2006 analysis from the same region, 2% of neonatal deaths were reported to be due to such infanticide [34]. However our data suggests that since 2007 this practice has stopped. We speculate that widespread education may have resulted in this practice ceasing.

From a practice standpoint, our research suggests that healthcare providers in this region of Ghana – including traditional birth attendants and local midwives who operate outside the facility setting – may benefit from additional training surrounding safe delivery, infant resuscitation, and clean delivery practices. This latter point is consistent with other research conducted by our group that found clean delivery practices were not always followed, especially outside formal healthcare settings [35]. Our results also emphasize the importance of educating women and other infant care providers, such as grandmothers, about the danger signs to watch for in the first few days following delivery.

In addition to the significant implications of our findings, we believe that this research has several important methodological strengths. The first is that this study is a prospective study which documented all pregnancies occurring over a 7-year period and followed them through to delivery, thereby reducing the chances of missing any neonatal death. This study includes data collected every four months on nearly 18,000 participants, as well as rigorous study oversight and the use of experienced study personnel to collect and analyse the data [29].

Nonetheless, this study has several limitations worthy of discussion. First, birth weight data was not available for the neonatal deaths recorded in this study. Frequently, birth weight is used as a proxy for gestational age, and both demonstrate similar interactions with neonatal deaths [4], [24], [25], [29], [36]. In this study, we used gestational age calculated based on the last menstrual period (LMP) and delivery date. This method is widely used in environments with limited technological resources, so even though recall of exact date of LMP may not be precise, we do not think this significantly biases the results. Second, as with any epidemiologic study, causality cannot be established from cross-sectional data. However, we found a strong association between prematurity, multiple births and first births and neonatal deaths. This relationship – while not definitively causal – provides valuable insight into risk factors for neonatal mortality among pregnant women in rural West Africa.

Another possible limitation of our study is the use of VA to determine the probable COD. Even though several studies have found VA to be a viable method to determine most COD[11], [37]–[41], it is not the gold standard. However, our rural West African setting did not allow for independent validation of verbal autopsy findings through laboratory, radiologic, microbiologic or post-mortem studies.

In summary, we found neonatal mortality rates are declining in the KND of rural northern Ghana, yet the majority of neonatal deaths occur during the first week of life. We found prematurity, multiple births and first births to be significantly associated with neonatal death, and our study found infections, prematurity, birth asphyxia and birth injuries to be the leading causes of neonatal deaths. Most of the deaths from infections were from septicaemia. Socioeconomic status and births from teenage mothers were not significant predictors of neonatal mortality in the study area, which may be attributable to improvements in health systems in this region of Ghana. Our study has important implications for research and practice, suggesting that mothers delivering for the first time and mothers of multiple births may require additional attention during the neonatal period, and providers may need additional training and resources to handle complicated and premature deliveries. Measure should also be put in place in health facilities to reduce neonatal sepsis by preventing and treating infections in mothers and providing a clean birth environment for save delivery in order to lower the chances of neonates contracting bacterial infections. Further research is needed to better understand the social, cultural, and logistical factors that are driving high mortality in the early days following delivery.

## References

[1] Black RE, Cousens S, Johnson HL, Lawn JE, Rudan I, et al (2010) Child Health Epidemiology Reference Group of WHO and UNICEF: Global, regional, and national causes of child mortality in 2008: a systematic analysis. Lancet 375(9730): 1969–1987. 20466419 10.1016/S0140-6736(10)60549-1

[2] World Health Organization (2006) Neonatal and Perinatal Mortality: Country, Regional, and Global Estimates.

[3] Baiden F, Hodgson A, Adjuik M, Adongo P, Ayaga B, et al (2006) Trend and causes of neonatal mortality in the Kassena-Nankana district of northern Ghana, 1995–2002. Trop Med Int Health 11(4): 532–539. 16553937 10.1111/j.1365-3156.2006.01582.x

[4] Engmann C, Jehan I, Ditekemena J, Garces A, Phiri M, et al (2009) Using verbal autopsy to ascertain perinatal cause of death: are trained non-physicians adequate? Trop Med Int Health 14(12): 1496–1504. 19799757 10.1111/j.1365-3156.2009.02395.x PMC3959775

[5] Zaidi AK, Huskins WC, Thaver D, Bhutta ZA, Abbas Z, et al (2005) Hospital-acquired neonatal infections in developing countries. Lancet 365(9465): 1175–1188. 15794973 10.1016/S0140-6736(05)71881-X

[6] Bryce J, Boschi-Pinto C, Shibuya K, Black RE (2005) WHO Child Health Epidemiology Reference Group: WHO estimates of the causes of death in children. Lancet 365(9465): 1147–1152. 15794969 10.1016/S0140-6736(05)71877-8

[7] Stanton C, Lawn JE, Rahman H, Wilczynska-Ketende K, Hill K (2006) Stillbirth rates: delivering estimates in 190 countries. Lancet 367(9521): 1487–1494. 16679161 10.1016/S0140-6736(06)68586-3

[8] Anonymous Stumbling around in the dark (2005) Lancet. 365(9476): 1983.

[9] Lawn J, Shibuya K, Stein C (2005) No cry at birth: global estimates of intrapartum stillbirths and intrapartum-related neonatal deaths. Bull World Health Organ 83(6): 409–417. 15976891 PMC2626256

[10] Oestergaard MZ, Inoue M, Yoshida S, Mahanani WR, Gore FM, et al (2011) United Nations Inter-Agency Group for Child Mortality Estimation and the Child Health Epidemiology Reference Group: Neonatal mortality levels for 193 countries in 2009 with trends since 1990: a systematic analysis of progress, projections, and priorities. PLoS Med 8(8): e1001080. 21918640 10.1371/journal.pmed.1001080 PMC3168874

[11] Edmond KM, Quigley MA, Zandoh C, Danso S, Hurt C, et al (2008) Aetiology of stillbirths and neonatal deaths in rural Ghana: implications for health programming in developing countries. Paediatr Perinat Epidemiol 22(5): 430–437. 18782251 10.1111/j.1365-3016.2008.00961.x

[12] Binka FN, Nazzar A, Phillips JF (1995) The Navrongo Community Health and Family Planning Project. Stud Fam Plann 26(3): 121–139. 7570763

[13] Binka FN, Bawah AA, Phillips JF, Hodgson A, Adjuik M, et al (2007) Rapid achievement of the child survival millennium development goal: evidence from the Navrongo experiment in Northern Ghana. Trop Med Int Health 12(5): 578–583. 17445125 10.1111/j.1365-3156.2007.01826.x

[14] Phillips JF, Bawah AA, Binka FN (2006) Accelerating reproductive and child health programme impact with community-based services: the Navrongo experiment in Ghana. Bull World Health Organ 84(12): 949–955. 17242830 10.2471/blt.06.030064 PMC2627578

[15] Chandramohan D, Shibuya K, Setel P, Cairncross S, Lopez AD (2008) Should data from demographic surveillance systems be made more widely available to researchers? PLoS Med 5(2): e57. 18303944 10.1371/journal.pmed.0050057 PMC2253613

[16] Setel PW, Whiting DR, Hemed Y, Chandramohan D, Wolfson LJ, et al (2006) Validity of verbal autopsy procedures for determining cause of death in Tanzania. Trop Med Int Health 11(5): 681–696. 16640621 10.1111/j.1365-3156.2006.01603.x

[17] Setel PW, Macfarlane SB, Szreter S, Mikkelsen L, Jha P, et al (2007) Monitoring of Vital Events: A scandal of invisibility: making everyone count by counting everyone. Lancet 370(9598): 1569–1577. 17992727 10.1016/S0140-6736(07)61307-5

[18] Engmann C, Ditekemena J, Jehan I, Garces A, Phiri M, et al (2011) Classifying perinatal mortality using verbal autopsy: is there a role for nonphysicians? Popul Health Metr 9: 42. 21819582 10.1186/1478-7954-9-42 PMC3160935

[19] Garenne M, Fontaine O (2006) Assessing probable causes of death using a standardized questionnaire: a study in rural Senegal. Bull World Health Organ 84(3): 248–253. 16583086 PMC2627283

[20] World Health Organization (2005) *World Health Organization International Statistical Classification of Diseases:* 10th Revision, 2nd Edition ed. Geneva: World Health Organization.

[21] Ghana Statistical Service (2009) *Ghana Demographic and Health Survey, 2008:* Accra: Ghana: Ghana Health Services (GHS) and ICF Macro.

[22] Liu L, Johnson HL, Cousens S, Perin J, Scott S, et al (2012) Child Health Epidemiology Reference Group of WHO and UNICEF: Global, regional, and national causes of child mortality: an updated systematic analysis for 2010 with time trends since 2000. Lancet 379(9832): 2151–2161. 22579125 10.1016/S0140-6736(12)60560-1

[23] Baqui AH, Darmstadt GL, Williams EK, Kumar V, Kiran TU, et al (2006) Rates, timing and causes of neonatal deaths in rural India: implications for neonatal health programmes. Bull World Health Organ 84(9): 706–713. 17128340 10.2471/blt.05.026443 PMC2627477

[24] Jehan I, Harris H, Salat S, Zeb A, Mobeen N, et al (2009) Neonatal mortality, risk factors and causes: a prospective population-based cohort study in urban Pakistan. Bull World Health Organ 87(2): 130–138. 19274365 10.2471/BLT.08.050963 PMC2636189

[25] Engmann C, Garces A, Jehan I, Ditekemena J, Phiri M, et al (2012) Causes of community stillbirths and early neonatal deaths in low-income countries using verbal autopsy: an International, Multicenter Study. J Perinatol 32(8): 585–592. 22076413 10.1038/jp.2011.154 PMC3922534

[26] Diallo AH, Meda N, Ouedraogo WT, Cousens S, Tylleskar T, et al (2011) A prospective study on neonatal mortality and its predictors in a rural area in Burkina Faso: can MDG-4 be met by 2015? J Perinatol 31(10): 656–663. 21372798 10.1038/jp.2011.6 PMC3183235

[27] Engmann C, Matendo R, Kinoshita R, Ditekemena J, Moore J, et al (2009) Stillbirth and early neonatal mortality in rural Central Africa. Int J Gynaecol Obstet 105(2): 112–117. 19201402 10.1016/j.ijgo.2008.12.012 PMC3972762

[28] Petterson B, Blair E, Watson L, Stanley F (1998) Adverse outcome after multiple pregnancy. Baillieres Clin Obstet Gynaecol 12(1): 1–17. 9930286 10.1016/s0950-3552(98)80036-9

[29] Engmann C, Walega P, Aborigo RA, Adongo P, Moyer CA, et al (2012) Stillbirths and early neonatal mortality in rural Northern Ghana. Trop Med Int Health 17(3): 272–282. 22175764 10.1111/j.1365-3156.2011.02931.x

[30] de Almeida MF, Alencar GP, Schoeps D, Novaes HM, Campbell O, et al (2011) Survival and risk factors for neonatal mortality in a cohort of very low birth weight infants in the southern region of Sao Paulo city, Brazil. Cad Saude Publica 27(6): 1088–1098. 21710006 10.1590/s0102-311x2011000600006

[31] Rahman S, Salameh K, Bener A, El Ansari W (2010) Socioeconomic associations of improved maternal, neonatal, and perinatal survival in Qatar. Int J Womens Health 2: 311–318. 21151678 10.2147/IJWH.S12426 PMC2990900

[32] Upadhyay RP, Dwivedi PR, Rai SK, Misra P, Kalaivani M, et al (2012) Determinants of neonatal mortality in rural Haryana: a retrospective population based study. Indian Pediatr 49(4): 291–294. 21992859 10.1007/s13312-012-0044-2

[33] Aborigo R, Moyer CA, Rominski S, Adongo P, Williams J, et al (2012) Infant nutrition in the first seven days of life in rural northern Ghana. BMC Preg Child 12(1): 76. 10.1186/1471-2393-12-76 PMC3490996 22857600

[34] Denham AR, Adongo PB, Freydberg N, Hodgson A (2010) Chasing spirits: Clarifying the spirit child phenomenon and infanticide in Northern Ghana. Soc Sci Med 71(3): 608–615. 20605304 10.1016/j.socscimed.2010.04.022

[35] Moyer CA, Aborigo RA, Logonia G, Affah G, Rominski S, et al (2012) Clean delivery practices in rural northern Ghana: A qualitative study of community and provider knowledge, attitudes, and beliefs systems. BMC Pregnancy Childbirth 12(1): 50. 22703032 10.1186/1471-2393-12-50 PMC3482570

[36] McClure EM, Wright LL, Goldenberg RL, Goudar SS, Parida SN, et al (2007) The global network: a prospective study of stillbirths in developing countries. Am J Obstet Gynecol 197(3): 247.e1–247.e5. 17826406 10.1016/j.ajog.2007.07.004 PMC2150563

[37] Edmond KM, Quigley MA, Zandoh C, Danso S, Hurt C, et al (2008) Diagnostic accuracy of verbal autopsies in ascertaining the causes of stillbirths and neonatal deaths in rural Ghana. Paediatr Perinat Epidemiol 22(5): 417–429. 18782250 10.1111/j.1365-3016.2008.00962.x

[38] Setel PW, Sankoh O, Rao C, Velkoff VA, Mathers C, et al (2005) Sample registration of vital events with verbal autopsy: a renewed commitment to measuring and monitoring vital statistics. Bull World Health Organ 83(8): 611–617. 16184280 PMC2626308

[39] Lawn JE, Osrin D, Adler A, Cousens S (2008) Four million neonatal deaths: counting and attribution of cause of death. Paediatr Perinat Epidemiol 22(5): 410–416. 18782248 10.1111/j.1365-3016.2008.00960.x PMC3428888

[40] Soleman N, Chandramohan D, Shibuya K (2006) Verbal autopsy: current practices and challenges. Bull World Health Organ 84(3): 239–245. 16583084 10.2471/blt.05.027003 PMC2627297

[41] Fottrell E, Byass P (2010) Verbal autopsy: methods in transition. Epidemiol Rev 32(1): 38–55. 20203105 10.1093/epirev/mxq003

